# Three Dimensional Quality Assessments of Applied Pelvic Binders

**DOI:** 10.29252/beat-070211

**Published:** 2019-04

**Authors:** Peyman Bakhshayesh, David Hullberg Risling, Anders Enocson

**Affiliations:** 1 *Imperial College London, Department of Cancer and Surgery, Karolinska Institute, Department of Molecular Medicine and Surgery, Stockholm, Sweden*; 2 *Karolinska Institute, Department of Molecular Medicine and Surgery, Stockholm Sweden*

**Keywords:** Pelvic binder, Pelvic trauma, Resuscitation, Hospital length of stay, 3D CT scan

## Abstract

**Objective:**

To assess the quality of applied pelvic binders using three dimensional computer tomography (3D CT).

**Methods:**

A local trauma registry was used to identify patients with pelvic fractures after high-energy trauma during 2011-2015. A 3D CT reconstruction was made from the initial trauma computer tomography images to assess the level of application, symmetricity of the binder and achieved fracture reduction. An acceptable application of the pelvic binder was deemed if it was at the trochanteric level, symmetric and minimized residual displacement.

**Results:**

We found 73 patients with a pelvic fracture and a pelvic binder on the initial trauma CT-scan. The mean (±SD) age of the patients was 46±17 years and 40% (n=29) were females. The median ISS score was 38 (IQR;29-50), the mean systolic blood pressure on arrival was 106±46 mmHg and the median GCS on arrival was 14 (IQR;7-15). We found that 59% (n=43) of the binders were correctly applied (symmetric at the trochanteric level, symmetrical and with acceptable residual displacement of the fracture). The 30-day mortality was higher in patients with non-correct application 17% (n=5/30) compared to patients with correct application of the pelvic binder 9.3% (n=4/43) however this was not statistically significant (*p*=0.562).

**Conclusion:**

A substantial number of patients had non-correct application of pelvic binders. Future studies using 3D technique are encouraged to further investigate clinical impacts of non-appropriate application of pelvic binders.

## Introduction

Pelvic ring fractures are uncommon and constitute about 3-8% of all fractures, but in major trauma cases (multiply injured patients and/or severe high-energy trauma) the incidence is increased to almost 20% [1]. These injuries often stem from high-energy blunt trauma like traffic accidents or fall from heights. Mortality in patients with pelvic fractures have been reported to be 7 to 47% [2] . Pelvic ring injuries might be lethal because of bleeding and it is therefore crucial to stop the haemorrhage as soon as possible before the patient’s physiology is impaired. By achieving pelvic reduction and stabilization it has been stated that clot formation is facilitated and the re-alignment of fractured bone surfaces helps to reduce the venous bleeding [3]. 

Immediate mechanical stabilization of pelvic fractures in poly trauma patients have therefore been advocated by several authors [4-9]. For this purpose application of pelvic binders (PBs) has become a routine procedure in polytrauma patients with pelvic fractures since the introduction by Vermeulen *et al*., [9]. The quickness and ease of application of PBs compared to previous methods has been described elsewhere [7]. According to the ATLS guidelines an external pelvic compression device should be applied if the patient is in shock and there is suspicion of a pelvic fracture, regardless of the fracture type [10]. In addition, some pelvic fracture patients might also benefit from a PB in the primary acute situation, regardless of the patients’ blood pressure. These are patients with an opening of, or a vertical displacement of, the pelvic bones [11]. It has even been proposed that application of a PB in a patient with a lateral compression type of fracture can be harmful [12].  Appropriate position of the PB has been shown to be important in different biomechanical studies to stabilize the disrupted pelvic ring and a PB placed over the greater trochanters is considered optimal [13, 14]. Following the manufacturer’s instructions the PB should be applied over the greater trochanters and also symmetrically applied to be as effective as possible [15]. However, there are only a few case-series or case-reports where PBs are used in clinical settings that assesses the physiological aspects [16]. To what we know, the impact of symmetric position of the PB is not studied in the literature. Another fact is that, apart from the SAM-sling, other types of PBs are almost radiolucent making the evaluation difficult. 

Our primary aim was to assess and report the quality of PB application in Sweden’s largest trauma centre (Karolinska University Hospital, Stockholm) in patients with high-energy pelvic fractures. Our secondary aim was to assess whether proper PB application did affect the outcome for trauma patients with pelvic fracture.

## Materials and Methods

 *Study design and study population*

 This was a registry study using a local trauma database at the Department of Orthopaedic surgery at the Karolinska University Hospital including high-energy pelvic fractures using information from the Swedish National Trauma Registry (SweTrau). At Karolinska University Hospital conducting initial CT scan following primary survey in poly-trauma setting is common practice. The database covers all traumatic pelvic ring injuries admitted to Trauma Centre Karolinska between 2011 and 2015. The inclusion criteria for the database were; a pelvic ring injury caused by a high-energy trauma [17], a Swedish ID-number, age ≥18 years and admittance by a trauma alert. From this database we identified, and included in the study, all patients with an applied PB by retrospective evaluation of the primary trauma CT-scans. Patients without PB on CT-scans were excluded.

*Assessment of pelvic binder application*

**Fig. 1:**
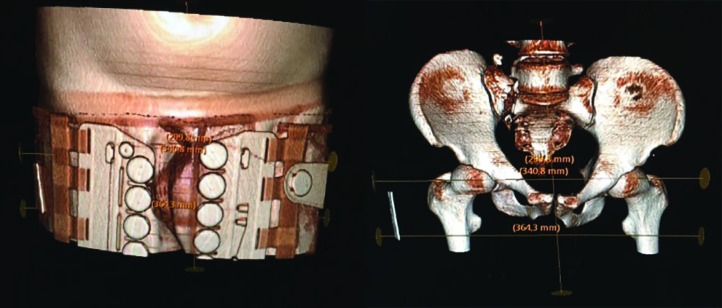
3D-CT images of a patient with the pelvic binder placed correctly – symmetrical and covering both greater trochanters

**Fig. 2:**
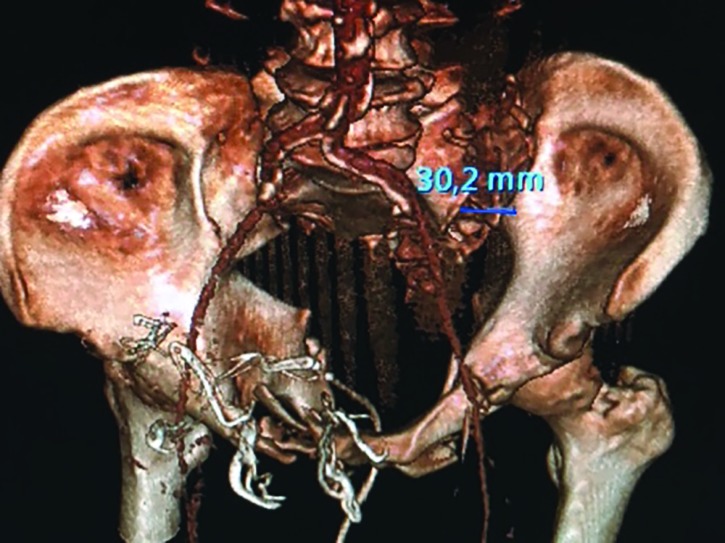
3D-CT image of a patient with residual displacement > 30 mm of the left sacroiliac-joint

 Three dimensional (3D) reconstructions of the primary trauma CT-scans using the Sectra® (Linköping, Sweden) software were made in all patients to assess the level of application, symmetricity of the PB in relationship to the pelvic ring and if there were any residual displacement of the pelvic fracture after PB application. We analysed whether the PB was applied at the level of the greater trochanters, or if it was lower or higher in this relationship. This was done by first scrolling in the 3D CT reconstruction of the frontal pelvic view to find a clear picture of the trochanters and then draw horizontal lines between the top limit of the greater trochanters and the lower limit of the minor trochanters. Then, by scrolling in the 3D layers to get a clear picture of the PB, assessment of whether the PB covered the area between the two horizontal lines could be made, if so it was deemed as trochanteric. If the lower part of the PB was above the inferior horizontal line the application was deemed as high and if the top part of the PB was under the superior horizontal line it was deemed as low. Assessment of the PB symmetry was made by comparing a line passing through the symphysis pubis and the mid of the sacral bone with the locking system of the PB. Symmetry was deemed if the midline was between the two halves of the locking system (Figure 1). Assessment of residual displacement of the fractures was conducted using criteria by Tornetta and Matta [18], and a residual displacement was defined as a displacement over 1 cm in any plane (Figure 2).

The data was assessed by a single observer (PB) and if there was any uncertainty the case was further reviewed (with the other authors) and agreement was achieved by consensus. The assessment of the fracture type was made by a pelvic surgeon (PB and/or AE) using the Young and Burgess classification and classified as; Lateral Compression (LC), Antero-Posterior Compression (APC), Vertical Shear (VS) or Combined Mechanism (CM) [19]. The first detected blood pressure was used as a marker of physiology. Patient was considered as in shock if the systolic blood pressure was less than 90 mmHg. Further data retrieved from the registry were mortality at 30 days, hospital length of stay, ICU length of stay, injury mechanism (motor vehicle accident, fall from high height or other), whether the injuries were intentional or not (meaning that the patient for example jumped from a building in a suicidal attempt), Glasgow Coma Scale (GCS) and Injury Severity Score (ISS). An appropriate application of the PB was deemed if it was at the trochanteric level, symmetric and with no residual displacement of the fracture. Non-acceptable application was deemed if any of these conditions was not fulfilled. We used the ATLS guidelines to define an unconditional indication for applying a PB as a patient with suspected pelvic injury in shock (systolic blood pressure less than 90 mmHg), regardless of the fracture type.

*Statistical Analysis*

                                                 Parametric data were analysed using the Student´s t-test and non-parametric data with the Mann-Whitney U test. Data with normal distribution was presented by mean and Standard Deviation (±SD) and median with Inter Quartile Range (IQR) was used to describe data with non-normal distribution. Categorical data were analysed with the Chi-square test. A *p*-value <0.05 was considered statistically significant. All tests were two-sided. The IBM SPSS Statistics software version 24 was used for statistical calculations.

## Results

From the local trauma registry 73 patients fulfilling the inclusion criteria were identified and included in the study. The mean age of all patients was 46±17 years and 29 (40%) patients were female. The median ISS score was 38 (IQR;29-50), the mean systolic blood pressure on arrival was 106±46 mmHg and the median GCS on arrival was 14 (IQR;7-15). Seventeen (23%) patients had a blood pressure under 90 mmHg and were therefore classified as in shock and defined as having an unconditional indication for PB application (Table 1).

**Table 1:** Patient, injury and fracture characteristics in all patients (n=73)

**Variable **	**Value **
**Age (years), mean± SD**	46±17
**Female gender, n= (%)**	29 (40%)
**ISS** a **, median (IQR)**	38 (29-50)
**Blood pressure (mmHg), mean± SD**	106±4.6
**GCS** b **, median (IQR)**	14 (3-15)
**Injury mechanism, n= (%)**	
**MVA** c **Fall from high height** **Other**	35 (48%)33 (45%)5 (7%)
**Injury Intention, n= (%)**	
**Intentional** **Non-intentional**	28 (38%)45 (62%)
**ICULOS** d ** (days), median (IQR)**	6.5 (1-18)
**HLOS** e ** (days), median (IQR)**	23 (12-41)
**Mortality 30-day all, n (%)** **Females** **Males**	9 (13%)4 (44%)5 (56%)
**Pelvic fracture type, n= (%)**	
**LC** f ** type I** **LC type II** **LC type III** **APC**^g^** type I** **APC type II** **APC type III** **VS**^h^ **CM**^i^ **Acetabulum only**	7 (10%)4 (5%)8 (11%)1 (1%)11 (15%)5 (7%)22 (30%)15 (21%)1 (1%)
**Patients in shock (BP< 90mmHg), n= (%)**	17 (23%)

a ISS: Injury Severity Score;

b GCS: Glasgow Coma Scale;

c MVA: Motor Vehicle Accident;

d ICULOS: Intensive Care Unit Length of Stay;

e HLOS: Hospital Length of Stay;

f LC: Lateral Compression;

g APC: Antero-Posterior Compression;

h VS: Vertical Shear;

i CM: Combined Mechanism; BP: Blood Pressure

*Injury and fracture characteristics*

 Thirty-five (48%) patients were injured in a motor vehicle accident, 33 (45%) had fallen from a height and five (7%) had other injury mechanisms. Twenty-eight (38%) of the injuries were intentional. Twenty-two (30%) patients had a VS fracture, 19 (26%) had an LC fracture, 17 (23%) had an APC fracture, 15 (21%) had a CM fracture and one (1%) had an isolated acetabular fracture.

*Binder application*

 The PBs used were a SAM-sling in seven (10%) patients and the other 66 (90%) patients had a T-POD. The level, with respect to the trochanters, of PB application was accurate in 59 (81%) patients, too low in 11 (15 %) and too high in three (4%). Nineteen (26%) patients had a non-symmetric application of the PB. Residual displacement of the fracture was seen in 30 (41%) patients. In the group of patients with a non-symmetric application 47% (n=9/19) had a residual displacement of the fracture, compared to 39% (n=21/54) in the group with symmetric application (*p*=0.6). A fully correct application of the PB, meaning symmetric at the trochanteric level and with no residual displacement, was seen in 43 (59%) of the patients (Table 2). Of the seven patients with a SAM-sling, three had an incorrect level of application. The 30-day mortality was 9.3% (n=4/43) in patients with correct application of the PB and 17% (n=5/30) in patients with non-correct application (*p*=0.5). 

**Table 2:** Analysis of pelvic binder application in all patients (n=73)

**Pelvic binder application**	**Number**	**%**
Greater trochanteric level	59	81
To high level	3	4
To low level	11	15
Symmetric application	54	74
Non-symmetric application	19	26
No residual fracture displacement	43	59
Residual fracture displacement	30	41
Correct application (right level, symmetric application and no residual fracture displacement)	43	59
Non-correct application (wrong level or non-symmetric application or residual fracture displacement)	30	41

GT; Greater trochanter level, Sym; Symmetrical application, Non-Sym; Non Symmetrical application ICULOS; ICU length of stay, HLOS; Hospital length of stay, A.; Acceptable, N.A.; Non-acceptable

**Table 3:** Comparisons between patients with different types of pelvic fractures

	**Patients with APC** f ** or VS** g ** type pelvic fracture (n=39)**	**Patients with LC** h ** type pelvic fracture (n=19)**	**P-value**
**Age (years), mean**	49±17	39±14	0.03^a^
**Female gender, n= (%)**	15 (38%)	11 (58%)	0.2
**GCS** b **, median (IQR)**	14 (8-15)	15 (4-15)	0.8
**ISS** c **, median (IQR)**	38 (33-50)	30 (17-50)	0.06
**HLOS** d ** (days), median (IQR)**	20 (7-28)	12 (9-35)	0.7
**ICULOS** e ** (days) median (IQR)**	5 (1-16)	2 (0-13)	0.1
**Mortality 30-day, n= (%)**	6 (15%)	1 (5%)	0.5

a Significant difference;

b GCS: Glasgow Coma Scale;

c ISS: Injury Severity Score;

d HLOS: Hospital Length Of Stay;

e ICULOS: Intensive Care Unit Length Of Stay;

f APC: Antero-Posterior Compression;

g VS: Vertical Shear;

h LC: Lateral Compression

## Discussion

Pelvic fractures are potentially lethal because of bleeding, and stabilization and reduction of the pelvic ring has shown to be of high importance. Different tools, both invasive like the pelvic c-clamp and external fixators, and non-invasive like the use of circumferential bed sheets and pelvic binders have been used but the application of pelvic binders has risen in popularity. To our knowledge this is the first study to use 3D reconstruction of CT-scans to report on quality of application of PBs.

*Placement of pelvic binder*

 When looking at correct placement of the PBs not more than 59% were correctly applied (symmetric application at trochanteric level and without residual displacement of the fracture). The PBs has been advertised as a non-invasive, quick and easy way of achieving stability and reduction of the pelvic ring. Placement of the PB at the correct level has in biomechanical cadaver studies been found to be important [16]. A retrospective study by Bonner *et al*. evaluated the actual level of application of PBs in a UK military field hospital [13]. The authors used plain X-ray on patients with an applied SAM-sling (includes a metal spring) to assess whether the binder was at the level of the greater trochanters or not. They found that 50% had an incorrect placement leading to an inadequate reduction of the pelvic fracture [13]. Bonner *et al*. showed that incorrect placement (with regards to the greater trochanters) of the PB resulted in an inferior reduction of the symphysis pubis in open-book fractures [13]. In their study only 50% of the patients had the PB applied at a correct level, whereas in our study we could see that the PB where applied over the greater trochanters in 81% of the patients. The reason for this difference might be that Bonners study examined the use of SAM-slings whereas in our study most PBs were from another brand; T-pod. The T-pod has a wider belt which possibly makes it easier to cover the greater trochanters. In our study, we had seven patients with SAM-sling and of these three had an incorrect level of application. Another reason for our better results might be that the importance of PB placement has been more advocated in the ATLS-training program since the Bonner’s study. A previous nationwide study based on a questionnaire from 2016 in Sweden has showed that 80% of the on-call trauma medical officers had clear awareness regarding the level of application of a PB [20]. Another factor to consider is that the study from Bonner *et al*. was based on trauma patients from a UK military field hospital in Afghanistan. Whether the placement of the PB was affected by a hostile and stressful environment is not mentioned but one might speculate that this could be a confounding factor. If a soldier is injured and wearing a uniform the first responders might not take the time to undress the patient making it more likely to not achieve a correct placement of the PB. Symmetricity of the PB application has, to our knowledge, not been addressed, studied or reported in the literature. One might speculate that a non-symmetric placed PB will not appropriately reduce the pelvic ring. Symmetric application is in fact advised by the manufacturer [15]. In our study 26% of the PBs were not symmetric. In the group of patients with a non-symmetric PB application 47% had a residual displacement of the fracture, compared to 39% in the group with symmetric application. This might indicate that symmetric application could be of importance even though the current material is too small to achieve statistical differences. We found no differences in 30-day mortality, hospital or ICU length of stay depending on the quality of application of the PBs. 

In patients with LC type fractures it has been proposed that a PB might cause an over-reduction of the fracture and thereby making the injury worse. We found no support for this when comparing patients with LC fractures and those with APC or VS fractures, and this is in line with a national survey from the United Kingdom [11].

*Strengths and limitations*

 The major strength of this study was the use of the 3D CT reconstruction since it gave more information and a more detailed picture of how PBs where applied compared to other studies based on plain radiographs. Another strength was that the study consisted of a consecutive series of patients from a major trauma centre. The major limitations of the study were its retrospective and observational design. Another limitation of our study was the population size as our study is clearly under powered. We could not take into consideration whether the PB was applied by a doctor in the trauma room or by the ambulance personnel in a pre-hospital setting since we did not receive this information from the trauma register or the patient medical records. However, all the patients were seen by a trauma team before the trauma CT-scan and if the PB was applied in an incorrect way the trauma team had then had the chance to adjust the position of the PB. We found potentials for improvement as a substantial number of the PBs were neither placed at the correct right level nor in a symmetric fashion. Further education via ATLS courses or locally arranged trauma courses with emphasis on appropriate application of PBs are recommended. Adjustment of pelvic binders in ER following arrival of the trauma patients with applied binders can be encouraged, especially in patients with ongoing physiological instability. We could demonstrate that the 3D analysis of trauma CT-scan was a feasible option to use for evaluation of applied PBs in trauma patients with a pelvic fracture.

## Conflict of interests:

The authors declare no conflict of interests.
